# Space invaders: Searching for invasive Smallmouth Bass (*Micropterus dolomieu*) in a renowned Atlantic Salmon (*Salmo salar*) river

**DOI:** 10.1002/ece3.6088

**Published:** 2020-02-15

**Authors:** Antóin M. O'Sullivan, Kurt M. Samways, Alysse Perreault, Cécilia Hernandez, Mark D. Gautreau, R. Allen Curry, Louis Bernatchez

**Affiliations:** ^1^ Canadian Rivers Institute University of New Brunswick Fredericton NB Canada; ^2^ Faculty of Forestry and Environmental Management University of New Brunswick Fredericton NB Canada; ^3^ Department of Biological Sciences University of New Brunswick Saint John NB Canada; ^4^ Institut de Biologie Intégrative et des Systèmes (IBIS) Pavillon Charles‐Eugène Marchand, Université Laval Québec QC Canada; ^5^ Department of Biology University of New Brunswick Fredericton NB Canada

**Keywords:** Atlantic Salmon, cross‐section, eDNA, invasive, qPCR, Smallmouth Bass

## Abstract

Humans have the ability to permanently alter aquatic ecosystems and the introduction of species is often the most serious alteration. Non‐native Smallmouth Bass (*Micropterus dolomieu*) were identified in Miramichi Lake *c*. 2008, which is a headwater tributary to the Southwest Miramichi River, a renowned Atlantic Salmon (*Salmo salar*) river whose salmon population is dwindling. A containment programme managed by the Department of Fisheries and Oceans, Canada (DFO) was implemented in 2009 to confine Smallmouth Bass (SMB) to the lake. We utilized environmental DNA (eDNA) as a detection tool to establish the potential escape of SMB into the Southwest Miramichi River. We sampled at 26 unique sites within Miramichi Lake, the outlet of Miramichi Lake (Lake Brook), which flows into the main stem Southwest Miramichi River, and the main stem Southwest Miramichi River between August and October 2017. We observed *n* = 6 positive detections located in the lake, Lake Brook, and the main stem Southwest Miramichi downstream of the lake. No detections were observed upstream of the confluence of Lake Brook and the main stem Southwest Miramichi. The spatial pattern of positive eDNA detections downstream of the lake suggests the presence of individual fish versus lake‐sourced DNA in the outlet stream discharging to the main river. Smallmouth Bass were later confirmed by visual observation during a snorkeling campaign, and angling. Our results, both eDNA and visual confirmation, definitively show Smallmouth Bass now occupy the main stem of the Southwest Miramichi.

## Graphical abstract

Smallmouth Bass were believed to be contained within Miramichi Lake. Our eDNA results suggested a high probability of escapement from Miramichi Lake into the lotic Southwest Miramichi. A visual observation of a Smallmouth Bass validated our results.

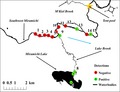

## 1 INTRODUCTION

Humans have the ability to permanently alter aquatic ecosystems via a myriad of pathways including habitat destruction (Hall, Jordaan, & Frisk, 2011; Liermann, Nilsson, Robertson, & Ng, 2012), modification of hydrological and thermal regimes (Moore, Spittlehouse, & Story, 2005; Zhang & Schilling, 2006), overharvesting of fish (Butchart et al., 2010; Taylor, Flecker, & Hall, 2006), and the introduction of non‐native species (Pister, 2001; Toussaint et al., 2018). The effects of introducing non‐native fish species has many effects on native species in aquatic environments (Jeschke et al., 2014; Sanderson, Barnas, & Rub, 2009) and the impacts can cascade through the entire ecosystem (Alexiades & Kraft, 2017; Eby, Roach, Crowder, & Stanford, 2006; Hilborn, 1999).

Smallmouth Bass (*Micropterus dolomieu*—SMB) are a cool water temperature species, with a high thermal tolerance (Brown, Runciman, Pollard, Grant, & Bradford, 2009). In Canada, the species is native to the Great Lakes Basin with the Acadian mountain range creating a natural migration barrier to the northeastern USA and eastern Canada (Curry, 2007). However, the species has been widely introduced across the east both illegally and via management programmes (Chaput & Caissie, 2010). In New Brunswick, Canada, SMB were introduced from Maine *c.* 1869 (Scott & Crossman, 1973) and populations are located primarily in the southeast in the Saint John and St. Croix rivers where they are naturalized (Curry & Gautreau, 2010). It is an apex predator and once established, has a high probability that it will never be extirpated (Brown et al., 2009).

In 2008, SMB were reported in Miramichi Lake, a headwater lake within the Miramichi River watershed and connected to the main stem Southwest Miramichi River via its outlet, Lake Brook, and these fish would have been introduced by humans (Valois, Curry, & Coghlan, 2009). The Miramichi River supports a world‐renowned Atlantic Salmon (*S. salar*) fishery. The species is an icon of the New Brunswick culture and Indigenous peoples as well as supporting a significant component of the NB economy (Gardner Pinfold, 2011). Atlantic Salmon populations are in a state of decline across much of eastern Canada (Veinott et al., 2018) and the Miramichi River is suffering the same declines (DFO, 2018). Consequently, the introduction of an apex predator into a warming watershed (Monk & Curry, 2009) with an already threatened native, cold‐water fish species is creating a high level of concern in the region (Chaput & Caissie, 2010). Introduced populations of SMB can have significant impacts on native fishes (Loppnow, Vascotto, & Venturelli, 2013) including salmonids (see reviews by Brown et al., 2009; Valois et al., 2009). Annual predation on outward migrating salmon smolts in the Pacific Northwest, USA, can be as high as 35% (Sanderson et al., 2009).

Environmental DNA (eDNA) is emerging is a reliable, efficient, and sensitive tool for identifying the presence and delimiting the distribution of aquatic species, and this is especially true when abundances are low (Franklin et al., 2018; McKelvey et al., 2016). eDNA is the DNA shed by an organism, for example, skin loss or feces, which persists in the surrounding environment (Wilcox et al., 2016). Water samples can be collected and analyzed, for example, via polymerase chain reactions (PCR), to search for target species (Goldberg, Sepulveda, Ray, Baumgardt, & Waits, 2013). The half‐life of eDNA or its detectability is dependent on UV‐B, temperature, and pH exposures in the host environment (Strickler, Fremier, & Goldberg, 2015), but even with these caveats, eDNA has proven effective in monitoring aquatic invasive species (Jerde, Mahon, Chadderton, & Lodge, 2011; Macissac, 2017). We used eDNA analyses to establish presence of SMB in Miramichi Lake and its potential distribution into the greater Southwest Miramichi River system.

## 2 METHODS

### 2.1 Study site

**Figure 1:**
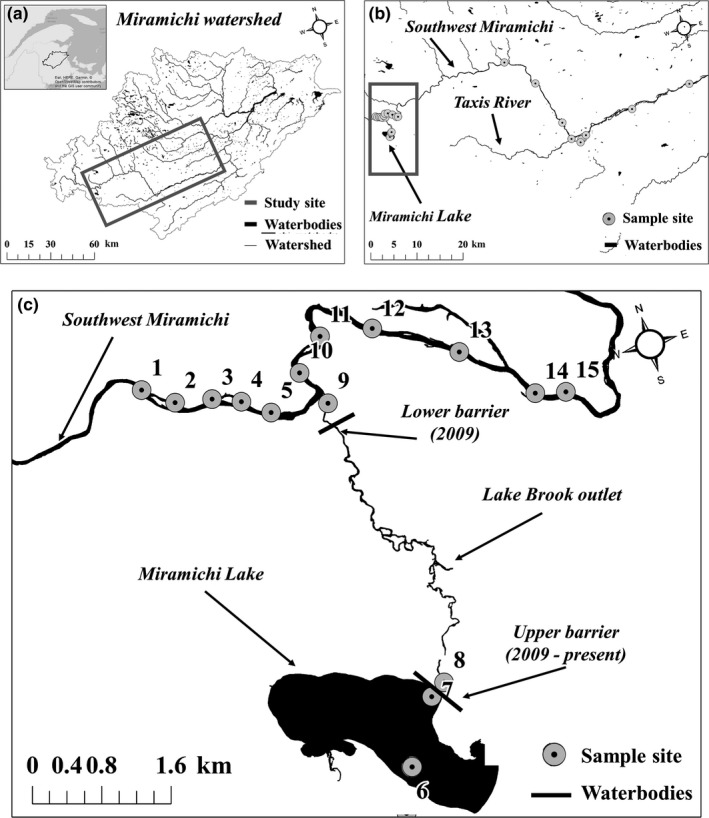
(a) Miramichi watershed and waterbodies, with study area delimited by the gray polygon. (b) Fine‐scale view of study area where the gray polygon denotes the Miramichi Lake, where Smallmouth Bass are known to exist. (c) Fine‐scale view of sampling effort in and around Miramichi, and spatial arrangement of containment barriers in effect from 2009 (lower barrier) to present (upper barrier)

The Miramichi River is located in New Brunswick, eastern Canada (Figure 1a). The watershed spans ≈14,000 km^2^ of mostly connected waterways (Cunjak & Newbury, 2005). Miramichi Lake is located at a headwater tributary (Lake Brook) to the Southwest Miramichi River (Figure 1b). Lake Brook (the lakes outlet) is ≈4.5 km long and is mostly free‐flowing with temporal changes in beaver dams. Since 2009, Fisheries and Oceans Canada (DFO) has installed a barrier net at the lake outlet for the period May to late October; and during 2009 a second net was installed in Lake Brook, ≈500 m above the confluence of Lake Brook and Southwest Miramichi (Figure 1c). The lake and upper Lake Brook section are assessed annually (netting and electrofishing), and the lower Lake Brook section—500 m above the confluence with the Southwest Miramichi (Figure 1c) has been assessed sporadically via angler and back‐pack electrofishing (in 2009, 2010, 2012, 2016, see Biron, 2018). All SMB are removed when captured, but SMB persist in the lake (young‐of‐the‐year are captured each year) and occasionally appear in the outlet stream (Biron, 2018). From 2010 to 2012 a “containment, control and eradication” plan was undertaken, and this was replaced by a containment, control and monitoring programme from 2013 to present day (Biron, 2018). Since 2015, there has been an increase in the number of SMB captured (Biron, 2018).

We collected eDNA samples from *n* = 26 sites within the lake, Lake Brook, and along the main stem of the Southwest Miramichi River (Figure 1b,c; Table 1). All samples were collected ≈10 cm below the water's surface. The lake samples were used to confirm our ability to detect SMB as they are known to be present in the lake (Biron, 2018). *N* = 2 samples were collected in Lake Brook; one sample was taken below the upper barrier and one was retrieved upstream of the brooks' mouth, to investigate if the eDNA signals were consistent in the brook below the barrier and upstream of the mouth of the brook (Figure 1). We collected five samples upstream of the confluence of Lake Brook and the main stem Southwest Miramichi River (Figure 1). We hypothesized that positive eDNA detection(s) upstream of Lake Brook would be indicative of SMB migration into the main stem, as eDNA can only flow downstream. *N* = 6 samples were collected below the confluence of Lake Brook and the main stem Southwest Miramichi River (Figure 1). This design was chosen to (a) establish the spatial variability of the eDNA signal along the main stem, and (b) search for potential SMB. We included the Taxis River because of unconfirmed reports of SMB occurrence in this system (C. Connell, New Brunswick Department of Energy and Resource Development, personal communication). Sites were sampled between the 24 August and 18 October 2017 (Table 1).

**Table 1:** Site number, date, and associated sampling reach, where “Sw M, Southwest Miramichi River” (Figure 1)

Site no.	Date	Reach
1	24 August	Sw M—Upstream of lake
2	24 August	Sw M—Upstream of lake
3^a^	24 August	Sw M—Upstream of lake
4	24 August	Sw M—Upstream of lake
5	24 August	Sw M—Upstream of lake
6	24 August	Miramichi Lake
7	29 September	Miramichi Lake
8	29 September	Lake outlet
9	18 October	Lake outlet
10^a^	18 October	Sw M—downstream of lake
11	18 October	Sw M—downstream of lake
12	18 October	Sw M—downstream of lake
13	18 October	Sw M—downstream of lake
14	18 October	Sw M—downstream of lake
15	18 October	Sw M—downstream of lake
16	1 September	Sw M—downstream of lake
17^a^	25 August	Sw M—downstream of lake
18	25 August	Sw M—downstream of lake
19	25 August	Taxis River
20	25 August	Taxis River
21	25 August	Taxis River
22	25 August	Taxis River
23	25 August	Taxis River
24	25 August	Sw M—downstream of lake
25	25 August	Sw M—downstream of lake
26	25 August	Sw M—downstream of lake

a Sites where field blanks were collected.

### 2.2 Field sampling

Field sampling followed the protocol established by Carim, McKelvey, Young, Wilcox, and Schwartz (2016). We prepackaged forceps, desiccant indicating silica gel beads, and cellulose nitrate membrane filters (Whatman—47 mm diameter/0.45 µl) housed in a filter funnel (Nalgene 145‐2020 analytical test filter funnel—250 ml capacity) in a sterile laboratory prior to field sampling. At each site, we pumped 3 L of water through a prepackaged filter using a GeoPump 2 Peristaltic Pump while wearing disposable gloves. Due to low flows throughout study period, sedimentation was minimal, and as such no filter clogging was observed during field sampling. Upon completion of filtration, samples were placed in prepackaged zip lock bags with silica gel beads. Samples were stored in an ice‐filled cooler and moved within 24 hr to a −20°C freezer at the University of New Brunswick, Fredericton. We collected three field blanks (Sites 3, 9, and, 21—Table 1) to test for contamination. This required filtering 3 L of deionized water using the same techniques and equipment outlined above. Samples were thereafter shipped frozen to University of Laval, Québec where they were stored at −20°C until DNA extraction.

eDNA was extracted using the protocol developed by Goldberg, Pilliod, Arkle and Waits (2011) Extracted eDNA was stored at −20°C until amplification by quantitative PCR (qPCR) method. For each extraction batch, an extraction negative control (no filter) was added and treated as the other samples to account for possible contamination (Carim et al., 2016).

### 2.3 qPCR analysis

In order to identify Smallmouth Bass, we utilized a segment of the mitochondrial DNA cytochrome *c* oxidase subunit 1 (COI) gene—fragment length 634 (April, Mayden, Hanner, & Bernatchez, 2011; Hebert, Ratnasingham, & deWaard, 2003). COI sequences for Smallmouth Bass were obtained from the bold system (Barcode of Life Database http://www.boldsystems.org/index.php/). A dilution series of synthetic DNA was run in each qPCR plate as a standard curve. The synthetic DNA is diluted from the resuspended synthetic stock (1,000,000,000 number of copies) in 1:10 dilutions to obtain 5 standard points that are run in triplicates in each plate (100,000, 10,000, 1,000, 100, and 10 number of copies). We also targeted related species known to be present in NB in order to rule out misclassification: Largemouth Bass (*Micropterus salmoides*) and Redbreast Sunfish (*Lepomis auritus*). These species are not known in the Miramichi River, but in the adjacent Saint John River (Curry & Gautreau, 2010). COI were utilized to develop primers and probes to maximize the number of mismatches between the targeted species and the related species using Geneious (https://www.geneious.com/) and verified using Primer Express 3.0 (Life Technologies). In addition, the primer blast tool (https://www.ncbi.nlm.nih.gov/tools/primer-blast/) was applied to verify the specificity of the amplification on other species that may be present in the same environment (Ye et al., 2012).

We tested specific primers and probes by qPCR method on DNA extracted from tissues of the targeted species, and the two related species (Largemouth Bass, and Redbreast Sunfish). The amplification was performed on the PCR 7500 Fast Real‐Time (Life Technologies) in a final volume of reaction of 20 µl—including 1.8 µl of each primer (10 µM), 0.5 µl of probe (10 µM), 10 µl of Environmental Master Mix 2.0 (Life Technologies), 3.9 µl of H_2_O, and 2 µl of DNA. This was completed under the following conditions: 2 min at 50°C, 10 min at 95°C, 50 cycles of 15 s at 95°C, and 60 s at 60°C. During qPCR, the degradation of the probe is accompanied by fluorescence and the level of fluorescence is measured in real time during each PCR cycle (Nolan, Huggett, & Sanchez, 2013). Thus, it is possible to determine the PCR cycle where the detection threshold of fluorescence is reached (*C*_T_). The greater the number of DNA copies, the faster the threshold is reached and a lower *C*_T_ value. The presence of DNA from the targeted species is confirmed when amplification is detected before the detection threshold of the fluorescence is reached (Arya et al., 2005). We applied two methods of specific primer tests: (a) FAST SYBR Green method for testing specificity and efficiency of primers; and (b) TaqMan method for testing specificity of primers and probe. Specificity and efficiency of all primer pairs were first tested with FAST SYBR Green method, once amplification of targeted species only was achieved, the TaqMan assay was performed with the species‐specific probes for further specificity enhancement. The efficiency of this primer set is 102.8%, and the limit of detection (>95% accurate positive detection) is 20 molecules per reaction. The specific sequences were as follows: MIDO‐COIF ACCATCTTCTCTCTTCATCTTGCG; MIDO‐COIR GCGAGGACTGGGAGCGATAA; and MIDO_COI_probe CCCTGTTTGTTTGGTCCGT. The amplicon length was 173 bp.

To analyze the collected eDNA samples, a TaqMan qPCR method was used with the addition of the SPUD to the reaction as well as a standard curve (Nolan, Hands, Ogunkolade, & Bustin, 2006). A total of four plates were used—plate 1a, plate 1b, plate 2a, and plate 2b. Plates 1a and 1b are replicates where the same samples have been used in three different wells (three replicates) in plate 1a and in three different wells (three replicates) in plate 1b for a total of six replicates per sample. DNA presence of each targeted species was tested on the six replicates for each eDNA sample and the negative extraction control. The SPUD is used as an internal positive control to evaluate the efficiency of reaction and to identify the presence of inhibitors in the samples (Nolan et al., 2006). The amplification was performed in a final volume reaction of 20 µl including 1.8 µl of each primer (10 µM), 0.5µl of probe (10 µM), 10 µl of Environmental Master Mix 2.0 (Life Technologies), 3.9 µl of SPUD, and 2 µl of DNA. This was performed under the following conditions: 2 min at 50°C, 10 min at 95°C, 50 cycles of 15 s at 95°C, and 60 s at 60°C.

For each targeted species, a synthetic DNA template of 500 base pairs (gBlocks, IDT) was designed from the COI sequence and used as a standard curve for quantification. Using the gBlocks, the detection threshold for each primer pair was determined by serial dilution until the fluorescence signal corresponding to one molecule was reached (Forootan et al., 2017). Finally, all qPCR results were quantified using a standard curve of known DNA quantities. The latter allowed us to quantify positive PCR amplification in number of molecules to quantify the relative quantity of DNA from the targeted species. The number of molecules per reaction of 20 µl (here after “number of molecules”) of the six replicates were averaged based only on positive amplifications. Finally, to confirm that positive detections represent actual targeted species, positive amplifications were sequenced via Sanger sequencing.

## 3 RESULTS

### 3.1 qPCR and detections

**Figure 2:**
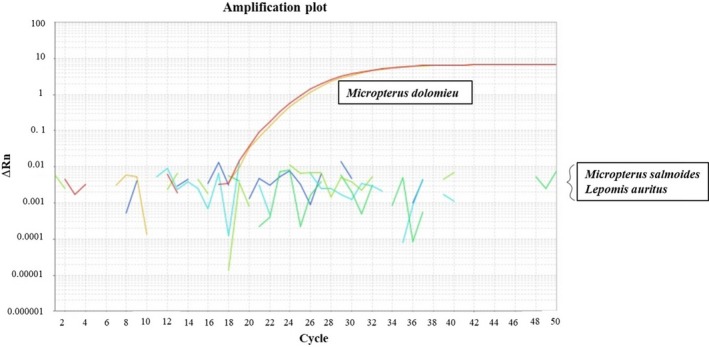
Results of specific amplification by qPCR for targeted species—Smallmouth Bass (*Micropterus dolomieu*)—and related species Largemouth Bass (Micropterus salmoides), and Redbreast sunfish (*Lepomis auritus*)—where “Cycle”, number of cycles located on the *y*‐axis, and “ΔRn”, fluorescence

After 50 cycles, the specific primers and probes amplified the targeted species, SMB, at ≈18 *C*_T_ (Figure 2). All extraction negative controls showed no positive amplification indicating the absence of contamination during DNA extraction. The three field negative controls (blank sample at Sites 3, 10, and 17) showed no positive amplification for the Smallmouth Bass (Table 2). Consequently, we can assert that the positive detection of Smallmouth Bass come from the sampled water and not from residual DNA which may have been inherent in field material. No amplification was detected for the related species, Largemouth Bass and Redbreast Sunfish (Figure 2). A fluorescence signal of 0.64 was chosen as best fit for all positive amplification across the four plates resulting in a *C*_T_ ≈40–41.5 (Wilson, Bronnenhuber, Boothroyd, Smith, & Wozney, 2014). This is within the range of acceptability outlined in Lockey, Otto, and Long (1998). Any amplification 41.5 > *C*_T_ was assumed to be either an artifact or an error and was disregarded.

**Table 2:** qPCR detection results for each site, where positive detection are bolded; “No. positive amplifications”, number of positive amplification; “*C*_T_ values” are the associated *C*_T_ values, and “No. molecules” are the number of molecules

Site no.	Reach	Detection	No. positive amplifications	*C*_T_ values	No. molecules
1	Sw M—Upstream of lake	Negative	0	—	—
2	Sw M—Upstream of lake	Negative	0	—	—
3^a^	Sw M—Upstream of lake	Negative	0	—	—
4	Sw M—Upstream of lake	Negative	0	—	—
5	Sw M—Upstream of lake	Negative	0	—	—
**6**	**Miramichi Lake**	**Positive**	**1**	**39.91**	**1.8**
**7**	**Miramichi Lake**	**Positive**	**2**	**40.27** **41.05**	**1.40** **1.02**
**8**	**Lake outlet**	**Positive**	**1**	**40.61**	**1.09**
**9**	**Lake outlet**	**Positive**	**1**	**40.8**	**1.21**
10^a^	Sw M—downstream of lake	Negative	0	—	—
11	Sw M—downstream of lake	Negative	0	—	—
12	Sw M—downstream of lake	Negative	0	—	—
**13**	**Sw M**—**downstream of lake**	**Positive**	**1**	**39.99**	**2.09**
14	Sw M—downstream of lake	Negative	0	—	—
**15**	**Sw M**—**downstream of lake**	**Positive**	**4**	**41.38** **41.38** **41.08** **39.72**	**0.59** **0.71** **1.00** **2.50**
16	Sw M—downstream of lake	Negative	0	—	—
17^a^	Sw M—downstream of lake	Negative	0	—	—
18	Sw M—downstream of lake	Negative	0	—	—
19	Taxis River	Negative	0	—	—
20	Taxis River	Negative	0	—	—
21	Taxis River	Negative	0	—	—
22	Taxis River	Negative	0	—	—
23	Taxis River	Negative	0	—	—
24	Sw M—downstream of lake	Negative	0	—	—
25	Sw M—downstream of lake	Negative	0	—	—
26	Sw M—downstream of lake	Negative	0	—	—

a Coupled field blanks.

**Figure 3:**
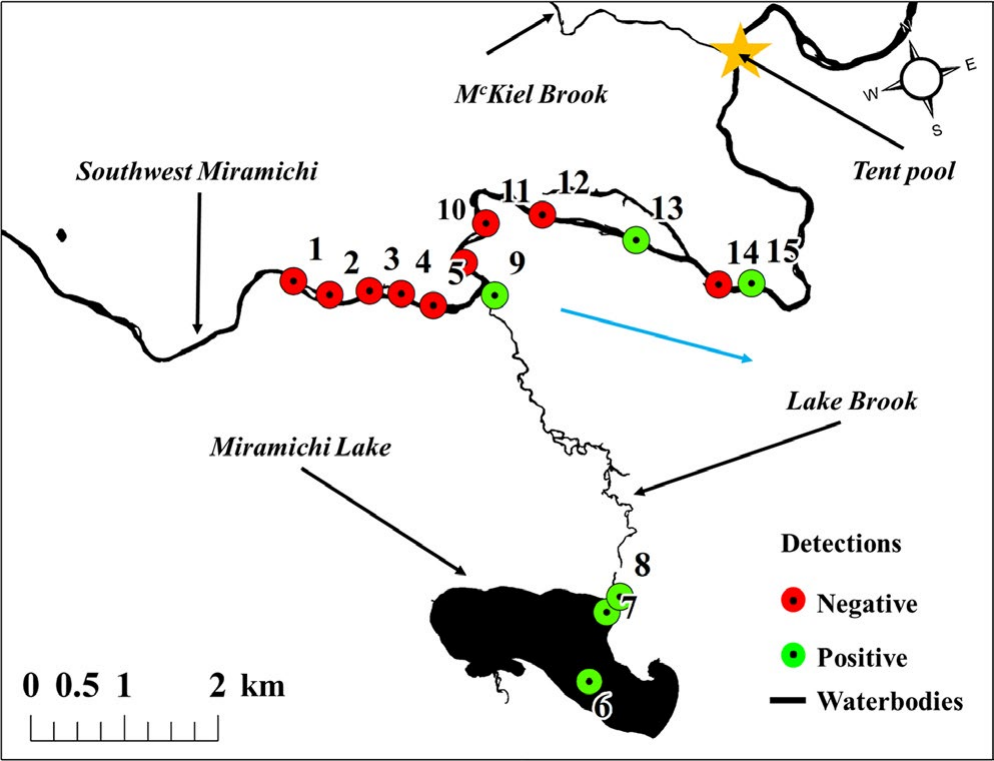
Detection results in and around Miramichi Lake, where the blue arrow defines flow direction, and negative DNA detections are red, while positives are green. The gold star highlights “Tent Pool” where a visual confirmation of a Smallmouth Bass occurred August, 2019

Positive amplifications were detected for Sites 6, 7, 8, 9, 13, 15 (Table 2, Figure 3), and these were located either within Miramichi Lake, Lake Brook, or in the main stem downstream of confluence with Lake Brook (Figure 3). *C*_T_ values for positive detections ranged from 39.72 to 41.38 (Table 2). Only one positive amplification was detected out of six replicates for Sites 6, 8, 9, and 13, two positives amplifications were detected for Site 7, and four positive amplifications were detected for Site 15 (Table 2). The total number of DNA molecules present in positive detections ranged from 0.59 to 2.50 molecules—Table 2. The highest number of molecules occurred at Site 13 (2.09 molecules) and 15 (2.50 molecules) and these were greater than the in‐lake samples (1.02–1.80 molecules). The results of the Sanger sequencing analysis (both forward and reverse strand) concluded all positive amplifications were SMB.

Although samples within Miramichi Lake were taken almost a month apart, DNA molecules were observed in both samples (Site 6:24 August, 2017—1.8 molecules; Site 7:20 September, 2017—1.02–1.40). Spatial variability of signal strength occurring in the main stem and downstream of the lake outlet was observed during sampling on 18 October, 2017.

## 4 DISCUSSION

Our primers and probes showed efficacy in amplifying SMB DNA. There were no indications of species misclassification and by testing against closely related species, that is, Largemouth Bass and Redbreast, we have accounted for concerns outlined in Wilcox et al. (2013) regarding sympatric taxa. Our results indicate that a SMB population persists in Miramichi Lake in agreement with field studies capturing SMB each year in the lake and occasionally in Lake Brook (Biron, 2018). The signal strength of the 2 eDNA samples in Lake Brook was relatively consistent—1 positive detection at each site (Table 2). There were no positive detections in the main stem reach immediately upstream of the confluence with Lake Brook, but detections occurred downstream within ≈3.2 km of the confluence. The increase in positive amplifications was observed 4.7 km downstream of the confluence with the Lake Brook (Site 15); however, the *C*_T_ values for 3 of 6 detections are on the cusp of our detection threshold, that is, 41.5 (*C*_T_ = 41.38, 41.38, 41.08). However, the Sanger sequence confirmed positive detections were SMB.

The patchiness of positive detections in the reaches downstream of the Lake Brook confluence with the main stem downstream area may be a function of DNA degradation or variances in flow regimes (Shogren et al., 2017). We sampled in the center of the main stem, and it is most likely the outflow of Lake Brook flows along the south bank, thus limiting our ability to detect an eDNA signal (Figure 3). Even so, UV‐b, temperature, and pH are also known to decay DNA (Strickler et al., 2015) and biotic processes such as biofilm abundance may also lead to eDNA degradation (Shogren et al., 2018). Jane et al. (2015) found that both low and high discharges alter DNA signal strength in running water and especially in turbulent reaches. These processes likely dilute and decay the DNA and therefore signals from the lake. However, there was an increase in the number of molecules observed with downstream distance from the confluence which is suggestive of SMB inhabiting this reach, that is, likely evidence of escapement from Miramichi Lake. Young‐of‐the‐year SMB have been captured in Lake Brook (its outlet—Biron, 2018; C. Connell, NB DERD, personal communication) despite the containment barriers used each year since 2008. In August 2019, we observed the first SMB in the mainstem of the river at ~8 km downstream of the outlet of Lake Brook and ~3 km downstream of Site 15 (Figure 3; Age 2–3; R.A. Curry, unpublished data). Since then, 10's of SMB have been angled from this reach, hence confirming our positive eDNA detections (M. Hambrook, Miramichi Salmon Association, personal communication).

Many factors will influence the degradation of eDNA (Shogren et al., 2018; Strickler et al., 2015) and which is complicated by the river's discharge (Jane et al., 2015; Wilcox et al., 2016). While we are learning more about these factors and interpreting/misinterpreting eDNA in lotic environments, we have minimized the potential for false negatives in our study by collecting field blanks to identify possible containments (Carim et al., 2016), tested similar species (Wilcox et al., 2013), and conducted the Sanger sequence to cross‐validated qPCR results (Knapp, Umhang, Poulle, & Millon, 2016). There remain pathways that may produce false positives (Roussel, Paillisson, Tréguier, & Petit, 2015; Wilcox et al., 2013). Hence, we suggest further, high density, testing targeting the reach where strong detections are suggested using both eDNA and efforts to capture the fish. With the current findings, we conclude that that SMB have most likely moved from the lake, where they were introduced, into the main stem of the Southwest Miramichi River.

## CONFLICT OF INTEREST

None declared.

## Data Availability

Data can be downloaded from https://doi.org/10.5061/dryad.ns1rn8pp4.
